# Highly elastic energy storage device based on intrinsically super-stretchable polymer lithium-ion conductor with high conductivity

**DOI:** 10.1016/j.fmre.2022.06.003

**Published:** 2022-06-18

**Authors:** Shi Wang, Jixin He, Qiange Li, Yu Wang, Chongyang Liu, Tao Cheng, Wen-Yong Lai

**Affiliations:** a State Key Laboratory of Organic Electronics and Information Displays (SKLOEID), Institute of Advanced Materials (IAM), School of Chemistry and Life Sciences, Nanjing University of Posts & Telecommunications, 9 Wenyuan Road, Nanjing 210023, China; b Frontiers Science Center for Flexible Electronics (FSCFE), MIIT Key Laboratory of Flexible Electronics (KLoFE), Northwestern Polytechnical University, Xi'an 710072, China

**Keywords:** Stretchable electronics, Flexible electronics, Flexible energy storage devices, Stretchable lithium-ion conductors, Flexible lithium-ion batteries

## Abstract

Stretchable power sources, especially stretchable lithium-ion batteries (LIBs), have attracted increasing attention due to their enormous prospects for powering flexible/wearable electronics. Despite recent advances, it is still challenging to develop ultra-stretchable LIBs that can withstand large deformation. In particular, stretchable LIBs require an elastic electrolyte as a basic component, while the conductivity of most elastic electrolytes drops sharply during deformation, especially during large deformations. This is why highly stretchable LIBs have not yet been realized until now. As a proof of concept, a super-stretchable LIB with strain up to 1200% is created based on an intrinsically super-stretchable polymer electrolyte as the lithium-ion conductor. The super-stretchable conductive system is constructed by an effective diblock copolymerization strategy *via* photocuring of vinyl functionalized 2-ureido-4-pyrimidone (VFUpy), an acrylic monomer containing succinonitrile and a lithium salt, achieving high ionic conductivity (3.5 × 10^−4^ mS cm^−1^ at room temperature (RT)) and large deformation (the strain can reach 4560%). The acrylic elastomer containing Li-ion conductive domains can strongly increase the compatibility between the neighboring elastic networks, resulting in high ionic conductivity under ultra-large deformation, while VFUpy increases elasticity modulus (over three times) and electrochemical stability (voltage window reaches 5.3 V) of the prepared polymer conductor. At a strain of up to 1200%, the resulting stretchable LIBs are still sufficient to power LEDs. This study sheds light on the design and development of high-performance intrinsically super-stretchable materials for the advancement of highly elastic energy storage devices for powering flexible/wearable electronics that can endure large deformation.

## Graphical abstract

**Figure:**
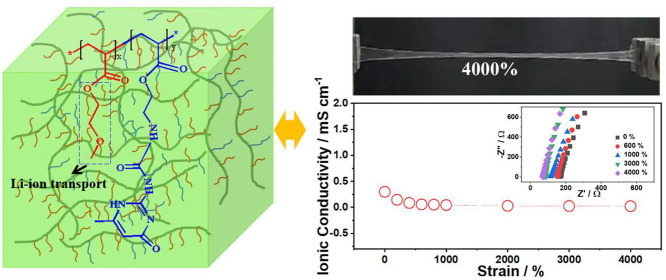

A highly elastic lithium-ion battery with strain up to 1200% is created based on an intrinsically super-stretchable polymer lithium-ion conductor. A diblock copolymerization strategy has been proved to be effective to construct the intrinsically super-stretchable conductive system, which achieves high ionic conductivity (0.35 mS cm^−1^ at room temperature) and can withstand large deformation (4560%). The results pave the way for design and development of high-performance intrinsically super-stretchable materials for the advancement of highly elastic flexible energy storage devices for powering flexible/wearable electronics that can endure large deformation.

## 1 Introduction

The growing demand for energy and the depletion of fossil fuels require the exploration of reliable, low-cost, and environmentally sustainable energy conversion and storage systems [1], [2], [3], [4], [5], [6], [7]. Lithium-ion batteries (LIBs) with features of lightweight, high energy density, and long life have been widely applied as the power source for electric vehicles, portable electronic devices, as well as large-scale energy-storage systems [8,9]. In recent years, stretchable energy storage devices such as stretchable supercapacitors [3,10], stretchable zinc-ion batteries [11,12], and LIBs [13,14] have attracted much interest. Among them, stretchable LIBs have attracted increasing attention due to their enormous prospects for powering flexible electronics, including artificial electronic skins [15,16], wearable devices [17,18], implantable medical devices [19,20], and stretchable displays [21]. Despite recent advances, it is still challenging to develop ultra-stretchable LIBs that can withstand large deformation under demanding conditions such as strenuous exercise.

Electrolytes are essential components of LIBs. The difficulty in constructing super-stretchable LIBs is that they rely on highly stretchable electrolytes as lithium-ion conductors with high and stable conductivity during large deformation. Most stretchable electrolytes show limited stretchability or unsatisfactory conductivity during large deformation [22], [23], [24], [25]. Currently, a stretchable LIB is typically assembled by using the poly(styrene-*b*-butadiene-*b*-styrene)-based electrolyte [26]. However, the electrolyte shows very poor stretchability, resulting in poor mechanical flexibility of devices. Although a recently designed supramolecular lithium ion conductor exhibits high stretchability [27], it unfortunately loses ionic conductivity under large deformation, also leading to low stretchability of LIBs. Obviously, the absence of the strain-tolerant electrolytes with both high stretchability and stable conductivity during large deformation is the key challenge to construct super-stretchable LIBs. On the other hand, although stretchable LIBs based on strain-engineering or rigid islands of device structures have been obtained [28], [29], [30], [31], the devices usually exhibit very limited stretchability (50%-100%). In addition, the above strategies significantly reduce the volume and weight energy density of the devices, which is typically unfavorable for flexible/wearable electronic devices. Hence, it is essentially critical to explore super-stretchable lithium-ion conductors simultaneously with high conductivity for the realization of highly stretchable LIBs.

In this contribution, we demonstrate a super-stretchable LIB (1200%) based on an intrinsically super-stretchable polymer electrolyte as the lithium-ion conductor with high ionic conductivity and excellent mechanical properties. A diblock copolymerization strategy is proposed to construct the intrinsically super-stretchable conductive system, which is created by fully exploring the synergistic effect between poly(ethylene glycol methyl ether acrylate) (PEGMEA), succinonitrile (SN), and poly(2-(3-(6-methyl-4-oxo-1,4-dihydropyrimidin-2-yl)ureido)ethylmethacrylate) (PUpyMA). The elastic networks (PEGMEA) wrap SN and lithium salt, leading to high ionic conductivity of 0.35 mS cm^−1^ at 30 °C. Meanwhile, the PUpyMA can further manage mechanical properties with high strength (over 120 KPa), superhigh stretchability (4560%), and electrochemical stability (over 5 V), thus realizing the synergistic regulation of ionic conductivity and mechanical properties in the solid electrolytes. To further demonstrate the utility of the polymer electrolyte in battery applications, LIBs were assembled using the as-prepared polymer electrolyte as the lithium-ion conductor, which showed excellent cycle performance and rate property. Stretchable LIBs based on the polymer conductor provide enough power to light LEDs, even in the repeated stretch and release states of 1200%. This study sheds light on the design and development of highly conductive and super-stretchable material systems for high-performance intrinsically stretchable power sources.

## 2 Experimental section

The related information of materials, synthesis of monomers and polymer electrolytes, and characterization can be found in Supporting Information. In addition, a Zennium Electrochemical workstation (ZahnerEnnium) was used for conducting electrochemical tests unless otherwise specified. [32], [33], [34] The ionic conductivity of PEU-x was calculated using σ=*l*/(*RS*) (*l*: thickness of electrolytes; *R:* bulk resistance; *S:* contact area between the stainless steel (SS) electrode and electrolytes). Charging or discharging time is 4 h at 0.25 mA cm^−2^ and 2 h at 0.5 mA cm^−2^ for galvanostatic cycling of Li/PEU-4/Li cell. To obtain the ionic conductivity of the electrolyte at deformation states, the film was stretched on a homemade stretching mold, followed by testing EIS perpendicular to the tensile direction and calculating. The electrochemical stability of PEU-x was measured by linear sweep voltammetry. The interface stability of PEU was evaluated by EIS using the Li/PEU/Li cell. The cell performance was evaluated on the CT-4008-5V10mA-164 battery testing system. In addition, the traditional LFP or LTO electrode was fabricated by casting slurry of LFP or LTO/PVDF/Super P (7:2:1) on Al foil. The mass loading is about 2 mg cm^−2^. All the electrochemical tests were conducted at 30 °C unless otherwise specified.

## 3 Results and discussion

**Fig. 1:**
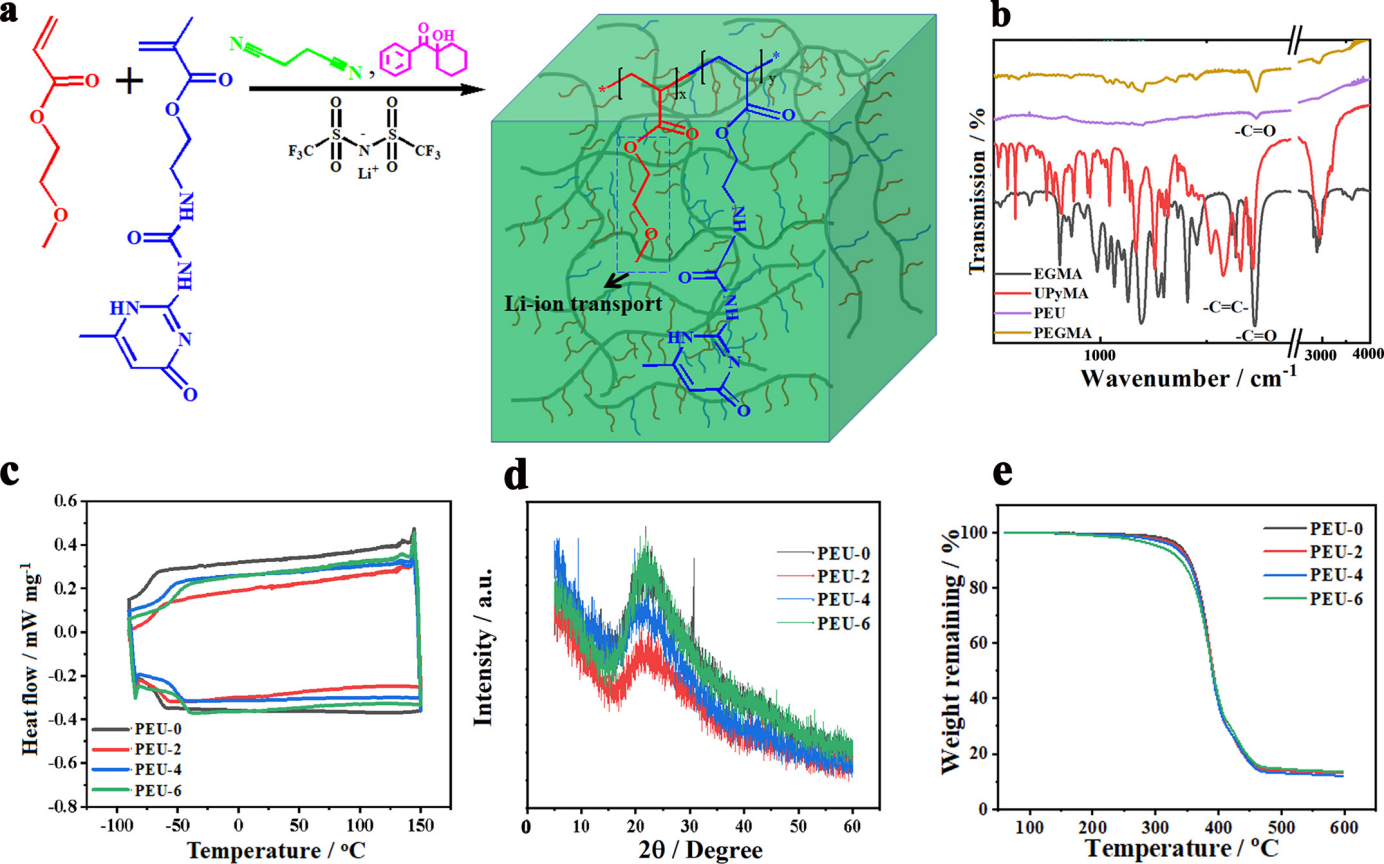
**The preparation and characterization of PEU.** (a) Synthetic route of PEU. (b) FTIR spectra of PEU precursors and PEU. DSC curves (c), XRD profiles (d) and TGA curves (e) of PEU-x (x = 0, 2, 4, 6), respectively.

Polymer electrolytes were prepared *via* the photocuring process. EGMEA/UpyMA, LiTFSI, and 1-hydroxycyclohexyl phenyl ketone acted as monomers, electrolyte salt, and photo-initiator, respectively (see the synthetic route in Fig. 1a and S1). Meanwhile, SN was employed to dissolve UpyMA and promote the transport of Li^+^. We systematically regulated the ratio of monomers for the preparation of polymer electrolytes to select the optimal monomer ratio by considering the mechanical tensile properties, elastic modulus, ionic conductivity and electrochemical stability of polymer electrolytes (see discussion below). Specifically, LiTFSI power and UpyMA were dissolved in SN to form a transparent liquid B_1_. Transparent liquid B_2_ containing EGMEA and photoinitiator was added into solution B_1_ to form the polymer electrolyte precursor, which was injected into a glass mold and cured by UV light irradiation for 10 h. The samples were named PEU-x (x = 0, 2, 4, 6) according to the mass percentage of UpyMA. EGMEA is crucial to endowing PEU with superior stretchability; The choice of using UpyMA can increase the mechanical strength and electrochemical stability of PEU; SN shows the ability to dissolve UpyMA and improve the electrochemical performance and stretchability of PEU.

**Fig. 2:**
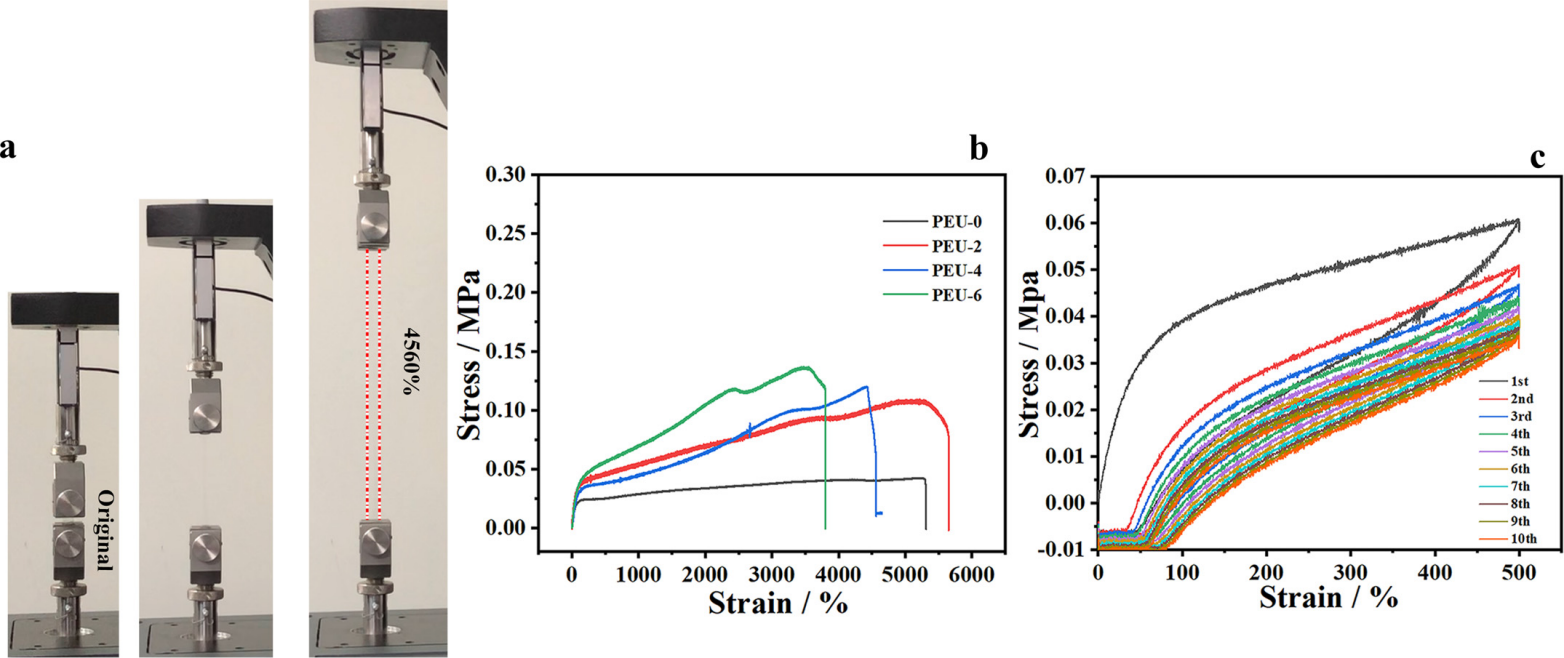
**Mechanical properties of PEU.** (a) Photographs of as-prepared PEU-4 at different tensile multiples. (b) Stress-strain curves of PEU-x (x = 0, 2, 4, 6). (c) Strain cycling of PEU-4. The strain rate is 50 mm min^−1^ for all the stress-strain tests.

FTIR spectrum of EGEMA and UpyMA in Fig. 1b show obvious characteristic peak of C=C double bond at ∼1640 cm^−1^. After polymerization, the characteristic peak belonging to the C=C double bond completely disappears (Fig. 1b), indicating the sufficient polymerization of active monomers. In addition, all the samples show peaks at 1733 cm^−1^, which belong to -C=O. The saturated hydrocarbon stretching vibration is also observed at 1350 and 1456 cm^−1^ for all the samples. For UpyMA, the peaks at 1254 cm^−1^ and 1580 cm^−1^ are the C—N and -NH characteristic peaks, respectively. These peaks are not obviously seen in FTIR spectrum of PEU due to the low content of UpyMA. Fig. 1c is the DSC curves of PEU-x (x = 0, 2, 4, 6). It shows that PEU-x possess *T_g_* in the range of -70 °C to -40 °C (-66 °C, -63 °C, -50 °C and -46 °C for PEU-0, PEU-2, PEU-4 and PEU-6, respectively), suggesting good motility of the polymer chain. In addition, it is seen that the *T_g_* values decrease with the increase of UpyMA content, suggesting that the mechanical strength of PEU-x samples is improved with the increase of UpyMA content (Fig. 2b). Moreover, no melting peak is seen in the curves, indicating the amorphous characteristics of PEU-x. It thus facilitates fast ion transport. The XRD profiles of PEU-x in Fig. 1d exhibit broad diffraction peak centered at ∼22^o^, further confirming the amorphous characteristics of PEU [35]. The TGA curves of PEU-x are shown in Fig. 1e, which exhibit ultra-high decomposition temperature (344 °C).

Photographs in Fig. 2a demonstrate superior stretchability and good transparency of PEU-4. Stress-strain curves of PEU-x (x = 0, 2, 4, 6) are shown in Fig. 2b to quantify the tensile capacity of PEU-x. PEU-0 exhibits superior stretchability, with average elongation at break of 4996% (according to Fig. S2 and Table S1), but low average failure strength of 0.062 MPa (corresponding average tensile modulus of 0.05 Mpa). The addition of UpyMA increases the average failure strength of PEU-x by nearly two times (0.099 Mpa, 0.11 Mpa and 0.13 MPa for PEU-2, PEU-4 and PEU-6, respectively), but the PEU-x still possesses excellent stretchability, with average elongation at break of 5464%, 4491% and 3746% for PEU-2, PEU-4 and PEU-6, respectively. The good mechanical performance of PEU-4 is further confirmed by bearing a weight of 0.15 kg (Fig. S3). It should be noted that decreasing the amount of UpyMA decreases the modulus of the polymer electrolyte (Fig. 2b), while further increasing the amount of Upy decreases the stretchability of the polymer electrolyte (Fig. 2b). Therefore, PEU-4 is optimal from the point of view of mechanical properties. The elastic recovery of PEU-4 was investigated *via* applying ten cycles of 0%-500% strain (Fig. 2c), which displayed good elastic recovery. Even after ten consecutive cycles, the residual stress of PEU-4 still maintained at ∼60%. In addition, the curves show hysteresis, indicating that there may be physical interaction such as H-bond between polymer chains (see FTIR spectrum in Fig. S4), leading to dissipation of strain energy. Similar results were also recorded for PEU-0, PEU-2 and PEU-6 (Fig. S5). We used ten consecutive stretches in an uninterrupted manner to demonstrate the good elasticity of the material, and in fact, the material is fully recoverable when the interval between two stretches is extended. This can be demonstrated more visually by the photos before and after stretching, as shown in Fig. S6, where the material can still return to its original state after being stretched over forty times.

**Fig. 3:**
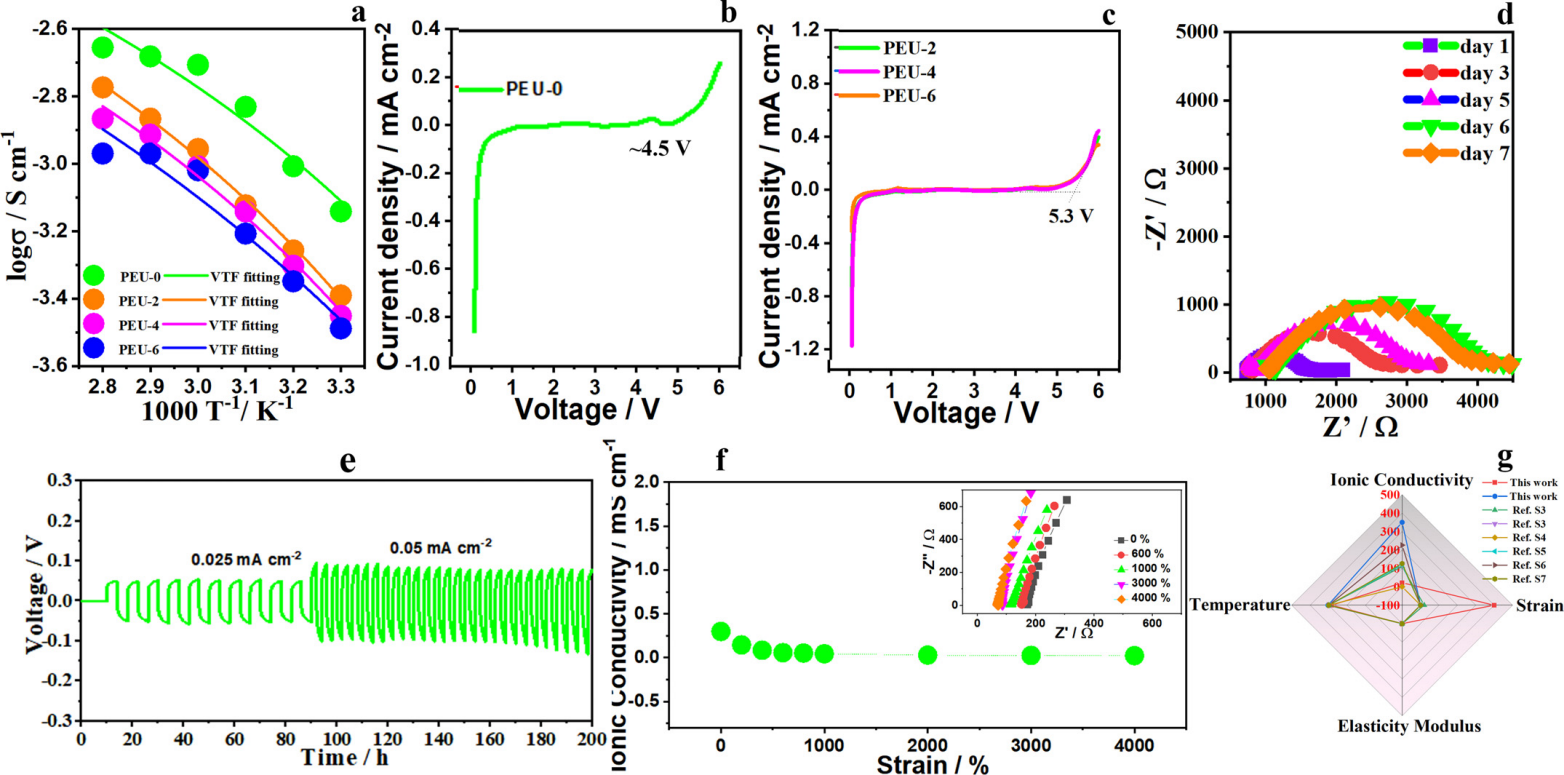
**Electrochemical performance of PEU.** (a) Ionic conductivity versus temperature of PEU-x (x = 0, 2, 4, 6). (b) LSVC of PEU-0. (c) LSVC of PEU-x (x = 2, 4, 6). (d) EIS of PEU-4 after different storage times. (e) Cycling stability of Li/PEU-4/Li cell under different current densities. (f) Stability of ionic conductivity during stretching. (g) A comparison between our work and reported stretchable electrolytes.

Apart from the features of superior stretchability and transparency, PEU-0, PEU-2, PEU-4, and PEU-6 exhibit high ionic conductivity of 0.72 mS cm^−1^, 0.41 mS cm^−1^, 0.35 mS cm^−1^, and 0.32 mS cm^−1^ at 30 °C (Fig. 3a), respectively. As the temperature rises, the ionic conductivity of PEU-x (x = 0, 2, 4, 6) increases and vice versa, due to the different contributions of different temperatures to the kinetic energy of the carriers, a phenomenon that has been widely observed in electronic conductors [36,37]. In addition, Fig. 3a shows that the ionic conductivity versus temperature from 30 °C to 80 °C generally obeys the Vogel-Tammann-Fulcher (VTF) relationship, which describes ion transport behaviors in the ion channels constructed by the polymer frameworks. According to the fitting results, low activation energies (*E_a_*) of 2.67 kJ mol^−1^, 3.45 kJ mol^−1^, 3.31 kJ mol^−1^, and 4.12 kJ mol^−1^ are obtained for PEU-0, 2, 4, and 6, respectively. Furthermore, taking PEU-4 as an example, SEM images in Fig. S7 present smooth microscopic surfaces, indicating good compatibility of the polymer matrix with lithium salt and SN.

The linear sweep volt-ampere curves (LSVCs) of PEU-0, PEU-x (x = 2, 4, 6) are shown in Fig. 3b and c, respectively. Compared with PEU-0 (∼4.5 V), PEU-x (x = 2, 4, 6) exhibit a wider electrochemical stability window (over 5 V), indicating that the addition of UpyMA facilitates the stability of the polymer electrolyte. Although the ionic conductivity of PEU-0 is higher than that of PEU-x (x = 2, 4, 6), its electrochemical stability is poor. In addition, PEU-x (x = 2, 4, 6) have good electrochemical stability and little difference in ionic conductivity, but PEU-4 has the optimal comprehensive mechanical properties. Based on the mechanical and electrochemical performances, PEU-4 is further studied in this work. As shown in Fig. 3d, EIS of Li/PEU-4/Li cell increases from day 1 to day 6 due to the formation of SEI film; a stable SEI film is formed, resulting in stable EIS after 6 days, demonstrating good interface stability of PEU with Li electrode. The symmetric Li/PEU-4/Li cell also exhibits steady plating/striping overpotential at current densities of 0.025 and 0.05 mA cm^−2^, respectively (Fig. 3e), further demonstrating good interface compatibility of PEU-4 with Li electrode.

For stretchable energy storage devices (SESDs), electrochemical properties of the electrolytes under large deformation, especially ionic conductivity, are the key to the good performance of SESDs under high stretch ratios. We measured the ionic conductivity of PEU-4 at 10 °C from 0% to 4000% strain. As shown in Fig. 3f, the initial conductivity of PEU-4 is 0.30 mS cm^−1^. Although the conductivity decreases to 0.044 mS cm^−1^ at 1000% strain, it should be noted that the test was conducted at 10 °C under 1000% strain; such value is still extremely high. The initial conductivity decreasing trend generally follows the shape-correlated function, because at small strains, polymer chains have not been globally aligned, and the deformation of main chain network with a weak mechanomodulation of ion conduction dominates electrical changes [38]. Moreover, the ionic conductivity is highly stable in the strain range of 1000% to 4000%. The inset of Fig. 3f presents the corresponding EIS traces at different stretchable states. Even ionic conductors such as hydrogels/ionogels have rarely reported possessing an elongation-at-break beyond 4000% strain while maintaining a satisfied modulus of > 0.12 MPa as well as stable and high ionic conductivity under large deformation (0%-4000%) [39], [40], [41]. We compared our material with recently-reported stretchable electrolytes in terms of ionic conductivity (Fig. S8), elasticity modulus, and strain at given temperatures, as shown in Fig. 3g and Table S2. Our material presents outstanding performances according to the given parameters. The stable and high conductivity of PEU mainly comes from the synergistic effect between PEGMEA, SN, and PUpyMA. Particularly, PEGMEA containing Li-ion conductive domains can strongly increase the compatibility of the neighboring elastic networks.

**Fig. 4:**
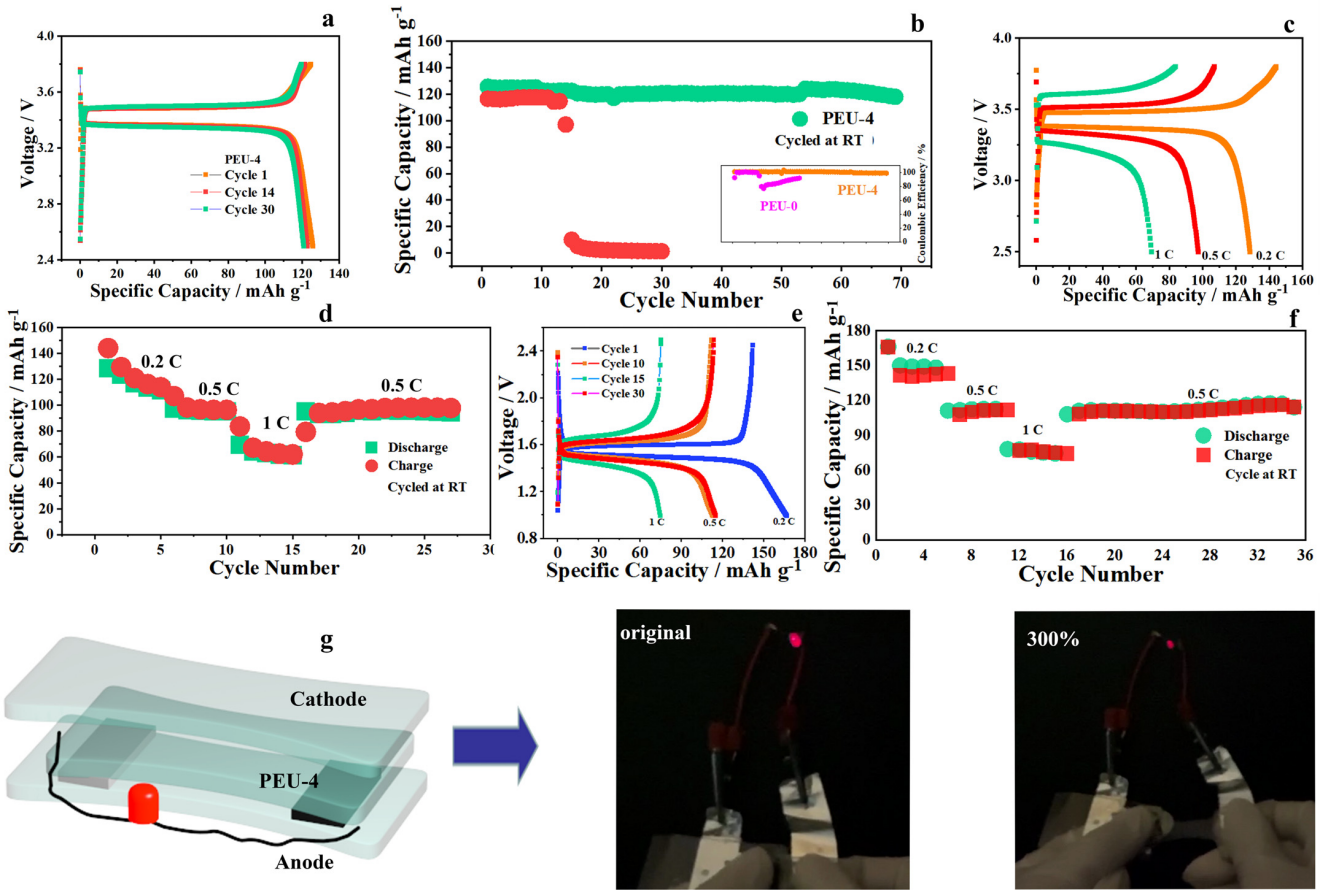
**Cell performance based on PEU-0/PEU-4.** (a) Charging/discharging curves of Li/LFP using PEU-4. (b) Cycle performance of Li/PEU-0/LFP and Li/PEU-4/LFP cells. Charging/discharging curves (c) and rate performance (d) of Li/PEU-4/LFP. Charging/discharging curves (e) and rate property (f) of Li/PEU-4/LTO cell. (g) Structure of stretchable battery, and photos of the cell under different stretched states.

The results for the solid-state batteries are shown in Fig. 4. The Li/PEU-4/LiFePO_4_ (LFP) cell displays high levels of reversibility and almost flat voltage platforms during charge and discharge at RT (Fig. 4a). The corresponding long-term cycling studies reported in Fig. 4b exhibit average Coulombic efficiencies of ∼100%, and stable cycling are obtained for 70 cycles. When the PEU-0 is applied in Li-LFP cells, large polarization is seen under the same condition (Fig. S9). In addition, both the battery capacity and Coulombic efficiency decay obviously (Fig. 4b). In addition, the long cycle performance of Li/PEU-4/LFP cell at 0.5 C is also confirmed. As shown in Fig. S10, the cell shows stable average capacity of about 110 mAh g^−1^ for 250 cycles. To further evaluate the performance of PEU-4, rate performance tests were performed using Li/PEU-4/LFP and Li/PEU-4/Li_4_Ti_5_O_12_ (LTO) cells, respectively. The charge-discharge curves of Li/LFP cell using PEU-4 under C-rates of 0.2, 0.5 and 1 C are shown in Fig. 4c. The overpotential increases (110, 250, and 360 mV at 0.2, 0.5 and 1 C, respectively) with the increase of current density due to the polarization effect. In addition, the cycling performance of Li/LFP cell using PEU-4 at the corresponding C-rates (Fig. 4d) demonstrates its good cyclic reversibility, with discharge capacity reaching 130 mAh g^−1^, 100 mAh g^−1^, and 66 mAh g^−1^ at 0.2 C, 0.5 C, and 1 C, respectively. For Li/PEU-4/LTO cell (Fig. 4e and 4f), it delivers reversible capacities of 142 mAh g^−1^, 112 mAh g^−1^, and 75 mAh g^−1^ at rates of 0.2 C, 0.5 C, and 1 C, respectively, suggesting superior reversibility and fast lithium storage kinetics using PEU-4. During operation, the ability of batteries to handle deformation is a crucial indicator of their eventual application in stretchable electronics. In particular, the development of highly stretchable LIBs can meet the large deformation requirements of wearable electronic devices in extreme sports and other conditions. To demonstrate that the obtained intrinsically stretchable ionic conductor (PEU-4) can be applied in stretchable LIBs, a stretchable LIB was assembled based on LFP cathode, Li anode, and PEU-4 to power an LED. The device can be stretched in the range of 0%-1200% (Fig. S11). As shown in Fig. 4g, the red LED works well when the device is stretched to 300%. Even when the device has been stretched 12 times (Movie S1), the red LED stays on, highlighting the applicability of the unique polymer electrolyte for highly stretchable LIBs.

**Fig. 5:**
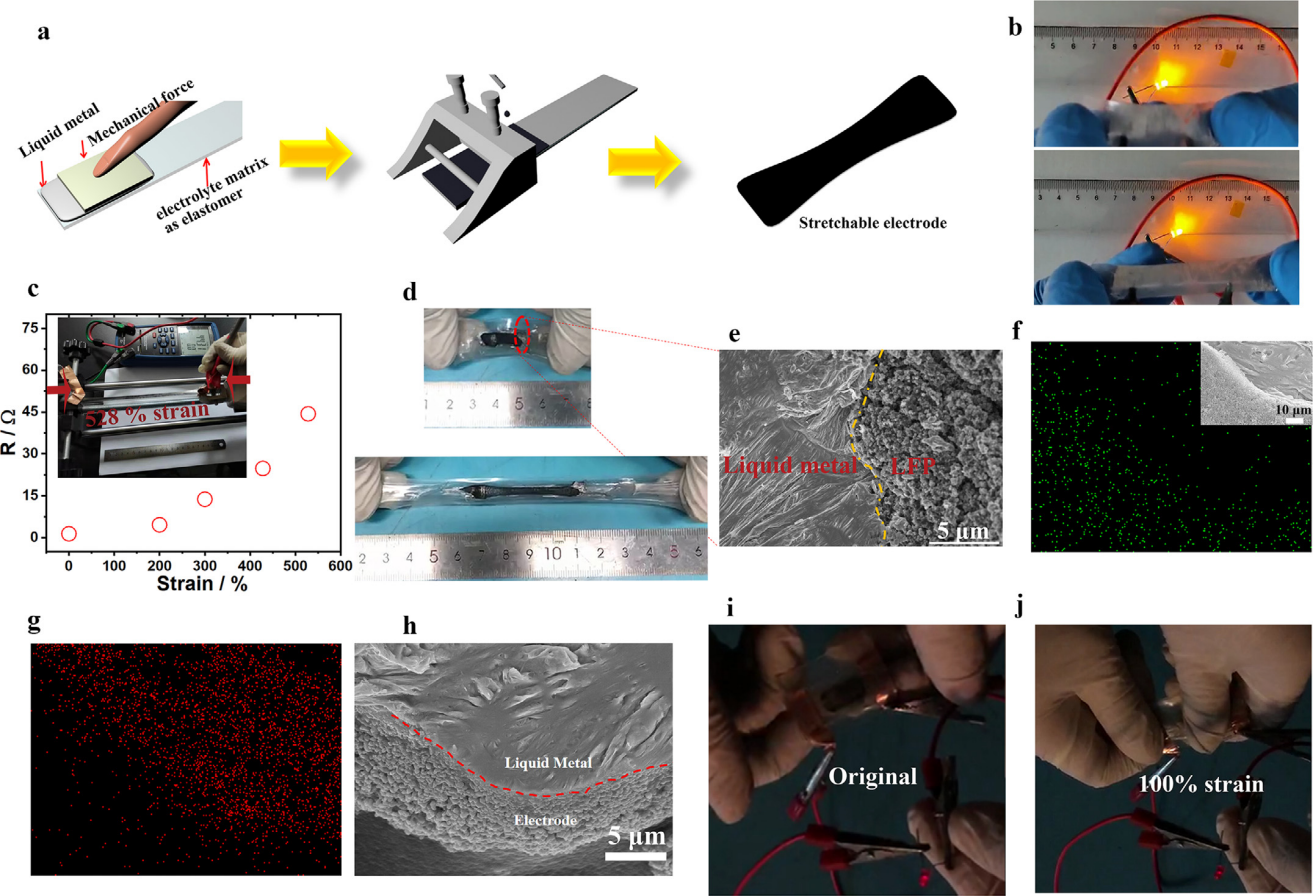
**Preparation and characterization of stretchable electrode.** (a) Fabrication route of the stretchable electrode. (b) SCC based on PEU-4 and liquid metal lights an LED at large deformation. (c) Relationship of resistance (R) and strain for the SCC. (d) Photos of electrode at original/stretched states. (e) SEM image of the electrode at the edge. EDS elemental mapping of P (f, inset shows the SEM image) and Ga (g) on the surface of the electrode. (h) Cross section of the electrode. A stretchable LIB powers an LED at original (i) and stretched states (j).

Intrinsically stretchable devices have many unique advantages, including low production costs and isotropic strain capacity [27]. However, inherent stretchability is difficult to achieve because the rigid conductive and electrode layers usually need to be embedded into an elastomer. The first task is to prepare a stretchable current collector (SCC). Liquid metals are eutectic alloys composed of metal atoms with a low melting point, which have practical value and inherent deformability. [42] Here, the Gallium indium tin alloy was cast onto PEU-4 by mechanical force to obtain an SCC (Fig. 5a). According to the SEM characterization, the liquid metal diffuses in the direction of the force in an orderly manner (Fig. S12). The as-prepared SCC has superior electronic conductivity that can light an LED at high tensile states (Fig. 5b). Even under the strain of 528%, the SCC has a low resistance of 44 Ω (Fig. 5c). We further cast electrode slurry on the SCC to achieve the intrinsically stretchable electrode (see Fig. 5a). To the best of our knowledge, such stretchable electrode is first reported for stretchable LIBs. As shown in Fig. 5d, the electrode can be reversibly stretched up to 400%. The SEM image of the electrode edge is shown in Fig. 5e; it is seen that the electrode material is in close contact with the liquid metal layer. The morphology remains unchanged even after repeated stretching, demonstrating a stable contact between the electrode and SCC. Fig. 5f (inset is the SEM of the electrode used to obtain the EDS) and Fid. 5g show the P and Ga maps, respectively. The uniform distribution P on the surface of SCC and Ga on the right of the electrode layer further reveals good contact of the electrode with SCC. In addition, the cross-section SEM image of the electrode further confirms the close contact between the SCC and the electrode material (Fig. 5h). At last, stretchable LIBs were fabricated using the as-prepared stretchable electrode, PEU-4, Li pieces, and encapsulated with PEU-4. As shown in Fig. 5i, j, the stretchable LIB (structure and photograph of the cell are shown in Fig. S13) can light an LED at original and ∼100% strain states. It is noted that the stretching of the device requires the matching of multiple functional layers, and further breakthrough is expected to be achieved by matching the highly stretchable electrolyte with advanced tensile electrodes. In addition, cyclic voltammetry (CV) test based on the as-prepared stretchable LFP-based cathode and Li anode from 2 V to 4 V was conducted. An obvious pair of cathodic/anodic peaks are seen in the CV curves (Fig. S14), corresponding to the lithiation/delithiation process. Such a result further highlights the applicability of liquid metal-based SCC in stretchable LIBs. Further improvement is on-going.

## 4 Conclusion

In summary, an effective diblock copolymerization strategy has been proposed to construct an intrinsically super-stretchable polymer lithium-ion conductor, which enables a super-stretchable LIB (up to 1200% strain). The resulting polymer conductive system exhibits a high RT ionic conductivity (0.35 S cm^−1^) and wide electrochemical window (5.3 V), while maintaining a high ionic conductivity over a large deformation range from 0% to 4000%. Additionally, the stretchable polymer conductor is a suitable elastic matrix for SCC. The prepared SCC (obtained by casting the liquid metal onto the surface of the polymer electrolyte) has a low resistance (∼44 Ω) at over 500% strain. In combination with the stretchable components, stretchable LIBs were created that still exhibited sufficient power to light LEDs when stretched to 1200% strain. The ultra-stretchable polymer lithium-ion conductor confirmed here offers an exciting way for fabricating ion-conducting materials with high ionic conductivity under ultra-high deformation to address the challenges of stretchable LIBs. We also expect to see a number of further applications in other areas, such as different kinds of intrinsically super-stretchable energy storage devices, soft robotics, scalable sensors, and other flexible/stretchable electronics.

## Declaration of competing interest

The authors declare that they have no conflicts of interest in this work.

## Appendix Supplementary materials

Image, application 1

Image, application 2
