# Supplementary material for: Highly elastic energy storage device based on intrinsically super-stretchable polymer lithium-ion conductor with high conductivity
Source: Fundam Res. 2022 Jun 18;4(1):140–6. doi: 10.1016/j.fmre.2022.06.003 (PMC11197603; doi:10.1016/j.fmre.2022.06.003)
Supplement: Supplementary file 2 [file mmc2.pdf]

Supporting Information

Highly Elastic Energy Storage Device based on Intrinsically Super-Stretchable Polymer Lithium-Ion Conductor with High Conductivity

Shi Wang,‡[a] Jixin He,‡[a], Qiange Li,[a] Yu Wang,[a] Chongyang Liu,[a] Tao Cheng,[a] and Wen-Yong Lai*[a,b]

[a] *State Key Laboratory of Organic Electronics and Information Displays (SKLOEID), Institute of Advanced Materials (IAM), Nanjing University of Posts & Telecommunications, 9 Wenyuan Road, Nanjing 210023, China*

[b]*Frontiers Science Center for Flexible Electronics (FSCFE), MIIT Key Laboratory of Flexible Electronics (KLoFE), Northwestern Polytechnical University, Xi'an 710072, China*

‡ Shi Wang and Jixin He contributed equally to this work.

*E-mail: *iamwylai@njupt.edu.cn*

Experimental section

Materials

Ethylene glycol methyl ether acrylate (EGMEA, 98%, Macklin), succinonitrile (99.7%, Macklin), 2-isocyanatoethyl methacrylate (98%, Aladdin), 6-methylisocytosine (98%, Aladdin), bis(trifluoro methane)sulfonimide lithium (LiTFSI, 99%, Aladdin) and 2-methyl-hydroxyphenol(photoinitiator, 98%, Aladdin) were used directly without other treatment. Gallium indium tin alloy was provided by Changsha Shengte New Materials Co. LTD.

Synthesis of UpyMA

UPyMA was synthesized as follows: 6-methylisocytosine (1.0 g, 8 mmol) was added into 25 mL of DMSO and stirred for 10 min at 150°C. After the solution was cooled to room temperature, 2-isocyanatoethyl methacrylate (1.32 g, 8.5 mmol) was added. The mixture was cooled by an ice bath and a white solid was precipitated, followed by collecting and drying the precipitate (under vacuum at 30°C for 10 h).

Synthesis of superior stretchable polymer electrolyte

2-(3-(6-Methyl-4-oxo-1,4-dihydropyrimidin-2-yl)ureido)ethylmethacrylate (UPyMA) was synthesized according to references.[1] UpyMA (0 wt%, 2 wt%, 4 wt%, 6 wt% according to the content of SN/LiTFSI) was dissolved in SN (0.64 g, contains 30 wt% LiTFSI), then EGMEA (0.8 g), photoinitiator (1 mol% of total double bond of the system) were added. The mixture was stirred for 0.5 h, and injected into a glass mold, followed by photopolymerization for 10 h (UVP CL-1000). Superior stretchable polymer electrolytes were obtained after further drying at 60°C for 10 h; the samples were named as PEU-x (x=0, 2, 4, 6) according to the contents of UpyMA.

Characterization

^{1}H NMR was obtained on a Bruker Ultra Shield Plus 400MHz NMR. FTIR spectroscopy (L1600400 Spectrum TWO DTGS) was used to record the structural information of monomers and PEU. The thermogravimetric analysis (TGA) of PEU was conducted on NETZSCH STA 2500 under N_2 (heating rate of 10°C min^{-1}), while the differential scanning calorimetry (DSC, Shimadzu DSC-60A equipment) was taken to investigate the thermal transition behaviors of the polymer electrolytes. The crystallization of PEU was further tested by XRD (Bruker D8 advance with a copper target, λ =1.54 Å. Instron 3300 electronic universal material testing system was used to test the mechanical properties of samples. Microstructures of PEU (sputtered with gold for 30 s), SCC and corresponding electrode were further characterized by Field Emission Scanning Electron Microscope (FESEM, Hitachi SU8010). SEM combined with EDS was used to investigate the element distribution of the electrodes.

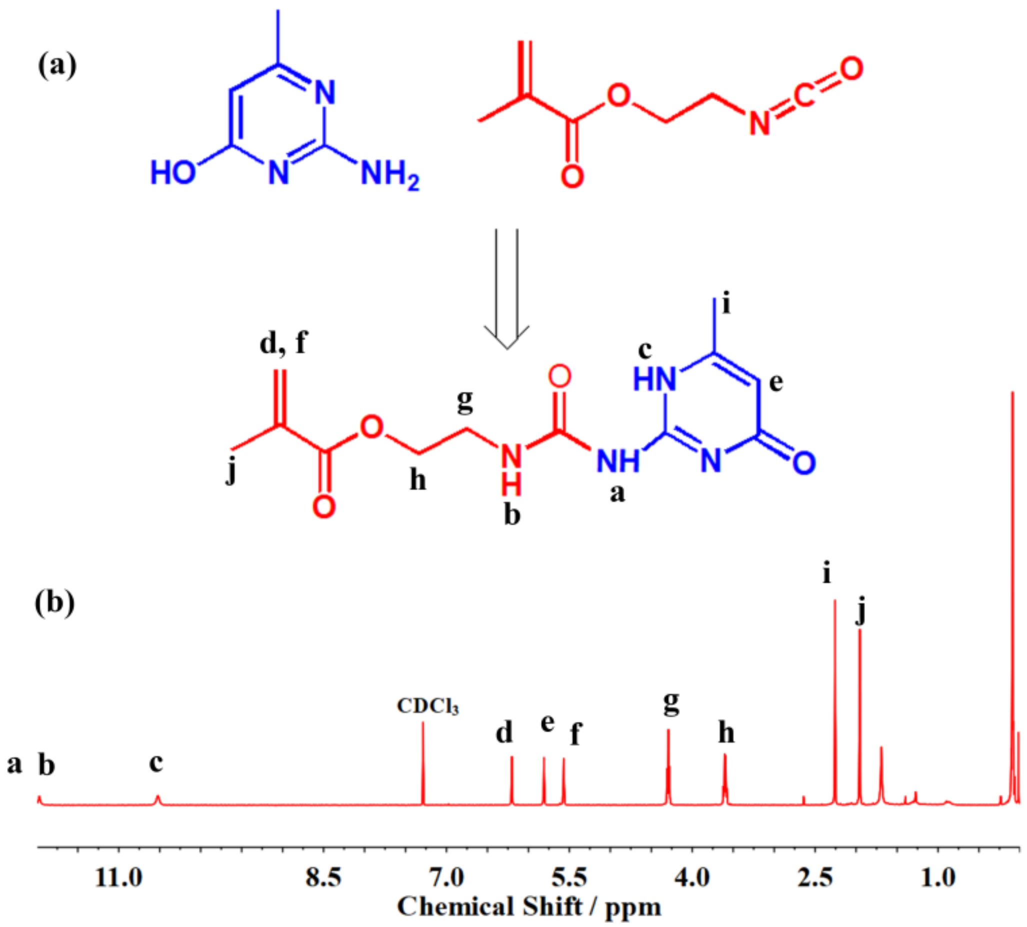

Figure S1. (a) Synthetic route of UpyMA. (b) ^{1}H NMR of UpyMA

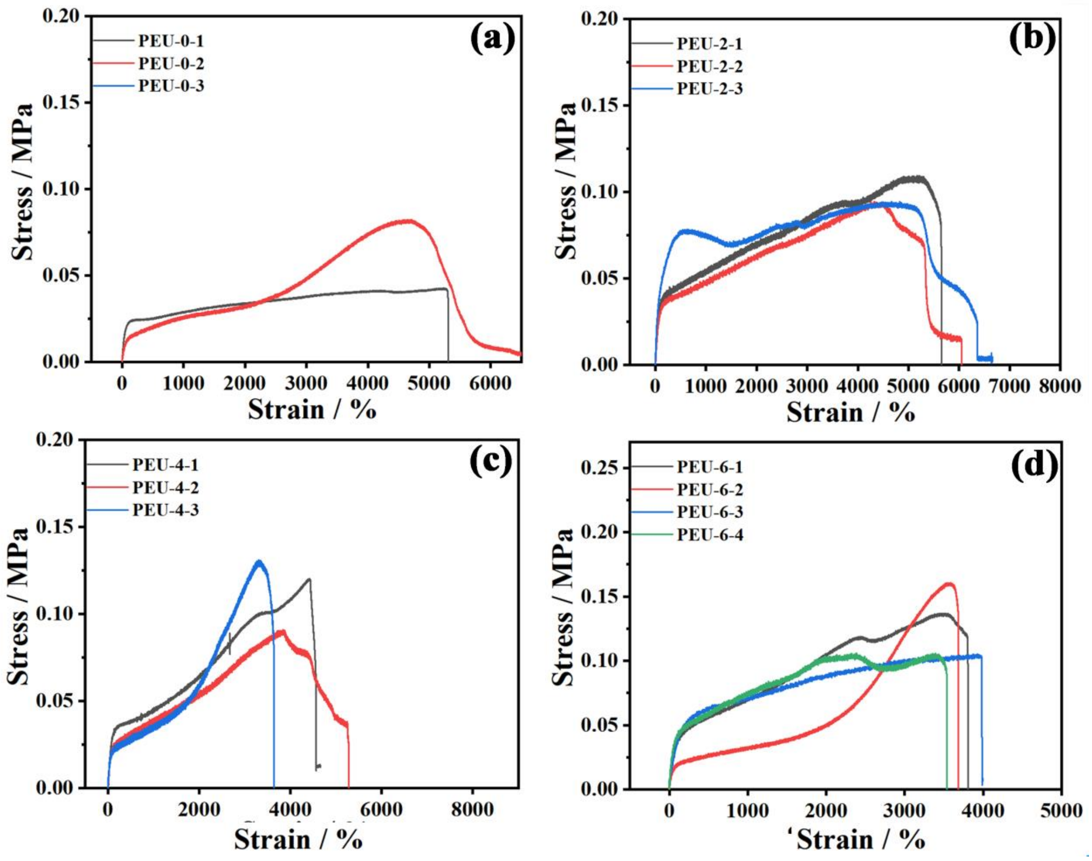

Figure S2. Stress-strain curves of PEU-x (x=0, 2, 4, 6).-

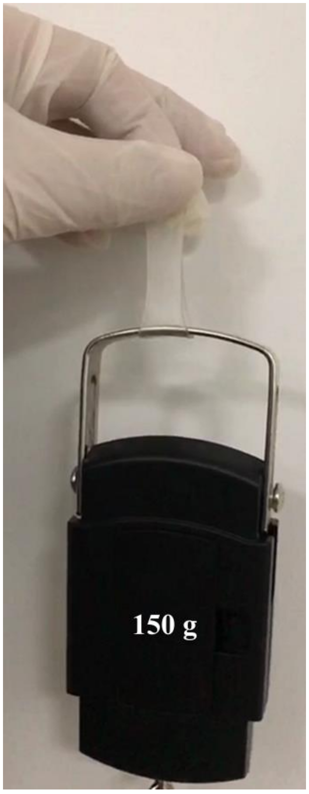

Figure S3. PEU-4 bearing 0.15 kg weight display

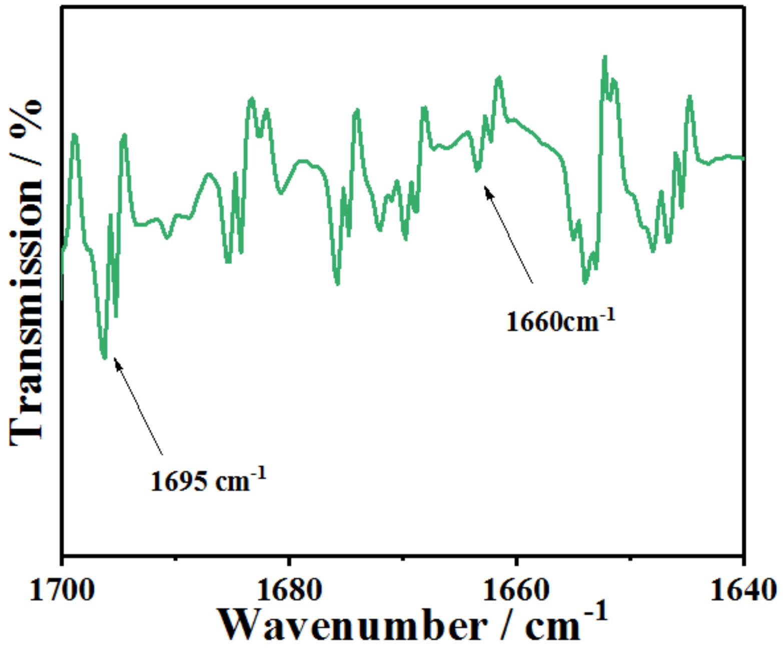

Figure S4. FTIR spectrum of PEU-4

C=O H-bonding in urea (1660 cm^{-1}) and C=O H-bonding in urethane (1695 cm^{-1}) from PEU-4 can be obviously observed, indicating the H-bond between polymer chains.

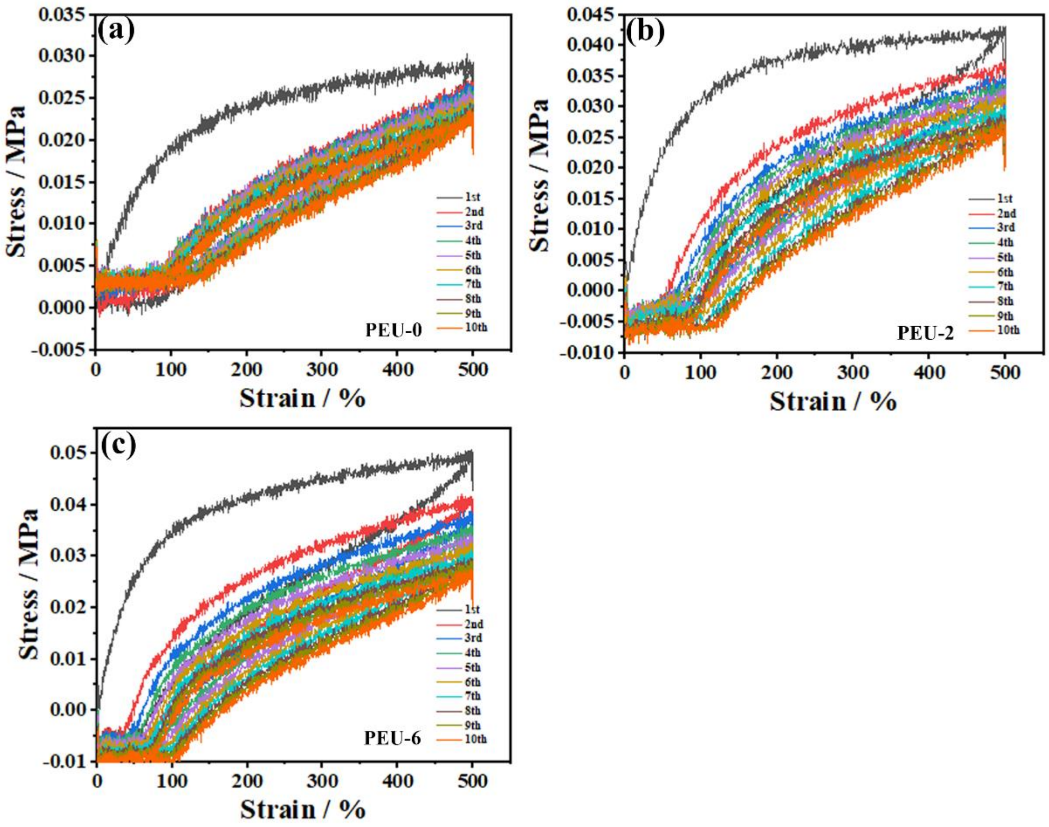

Figure S5. Strain cycling of PEU-0 (a), PEU-2 (b) and PEU-6 (c) at a rate of 50 mm min^{-1}.

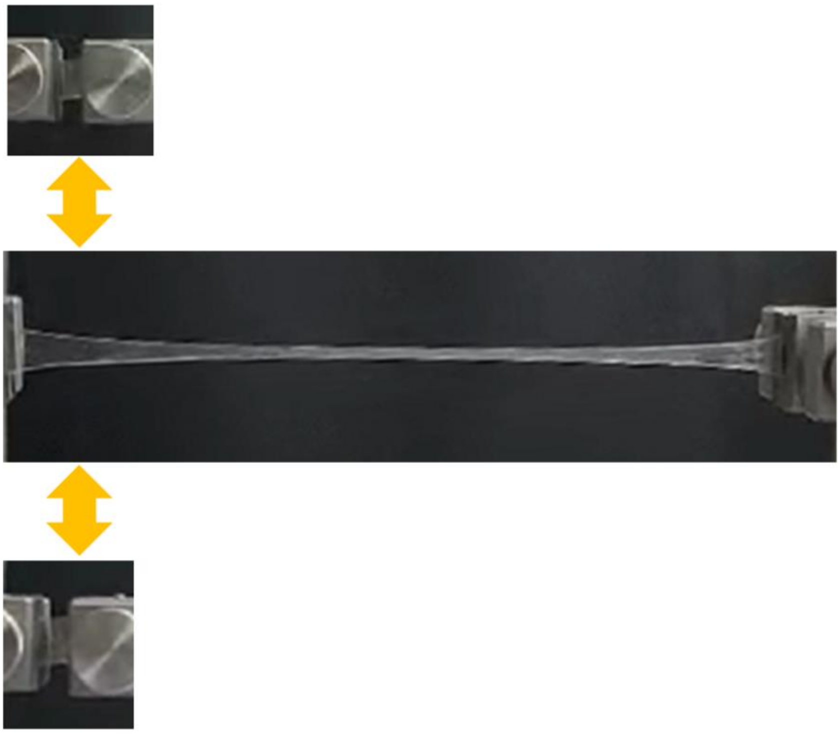

Figure S6. The resilience of PEU-4: The figures show the initial (top), stretched (middle) and recovered (bottom) states of PEU-4, respectively.

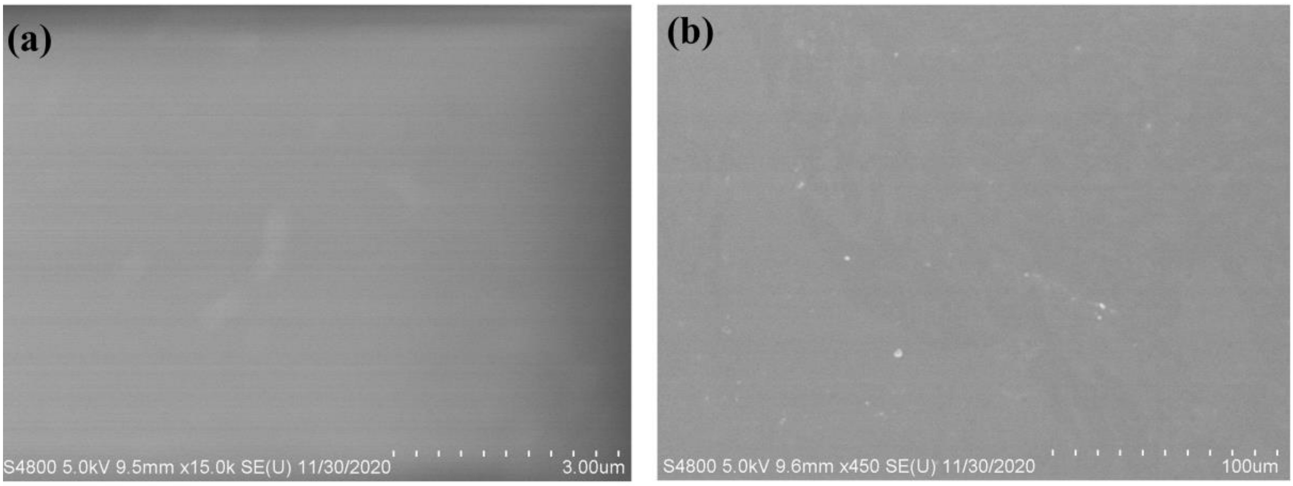

Figure S7. SEM images of PEU-4

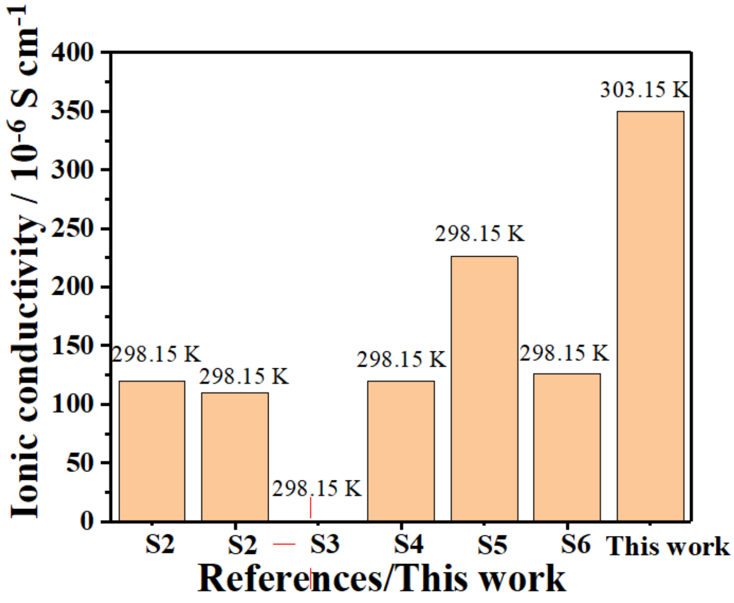

Figure S8. Ionic conductivity of PEU-4, which is compared with references.

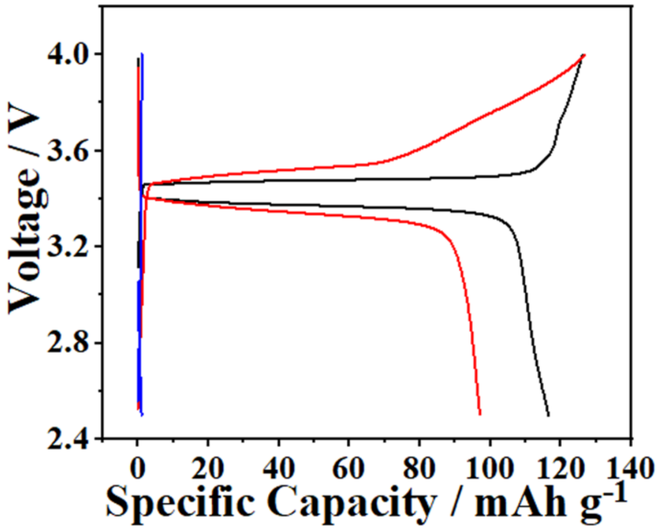

Figure S9. Charge-discharge curves of LFP/PEU-0/Li cell at 30°C

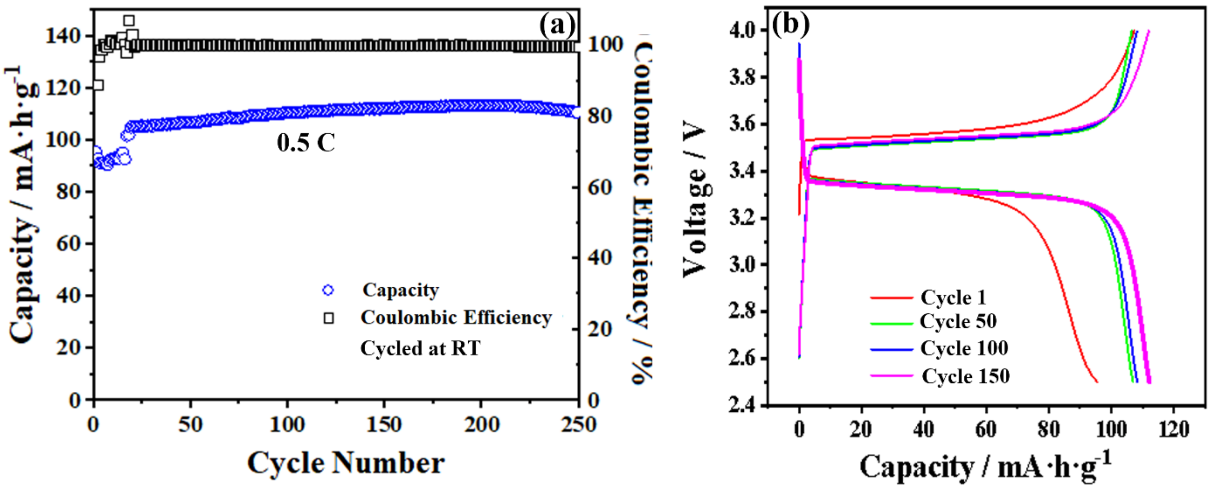

Figure S10. Cycle performance of (a) Li/PEU-4/LFP cell at 0.5 C and (b) corresponding charge/discharge curves.

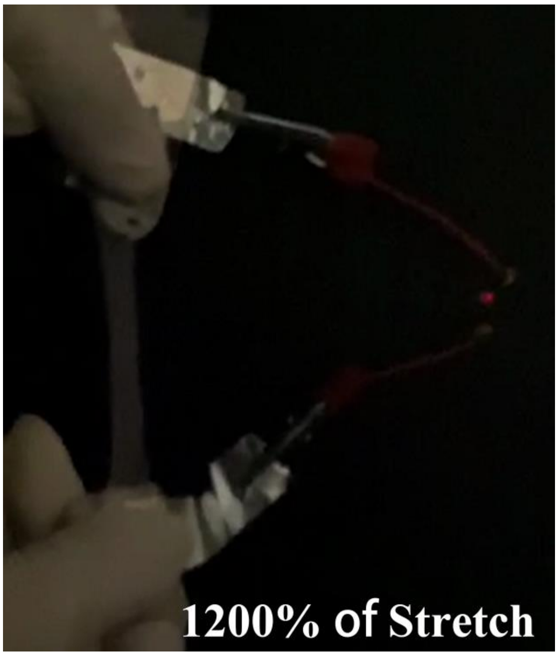

Figure S11. SLIB with strain of 1200%

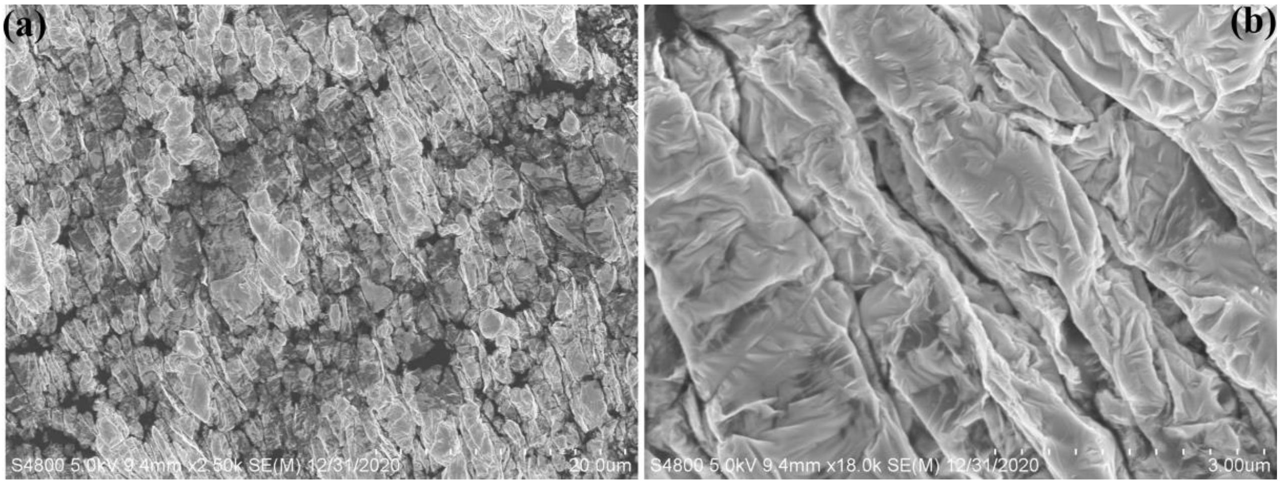

Figure S12. SEM images of liquid metal on PEU-4 at different magnifications

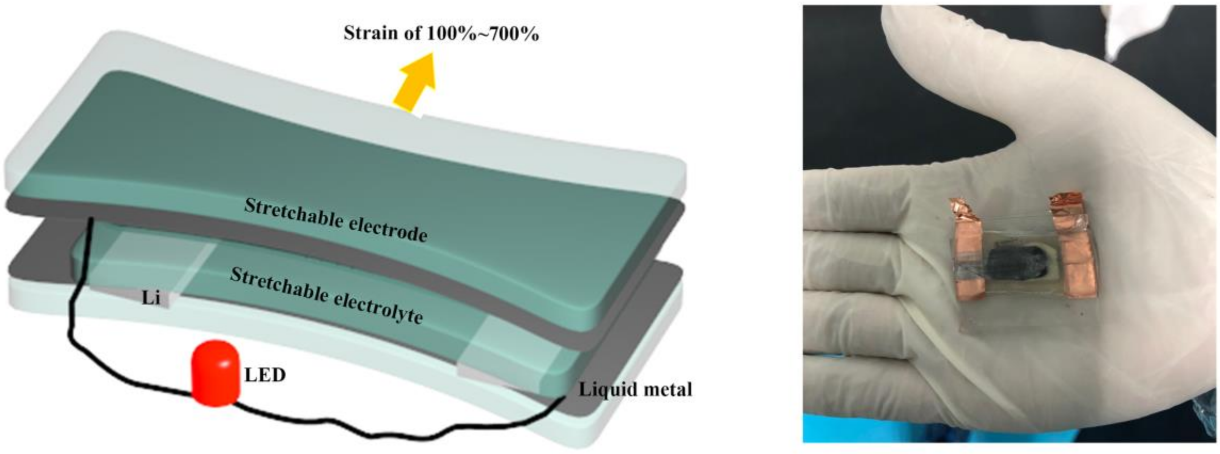

Figure S13. Schematic device structure and photograph of the stretchable LIBs.

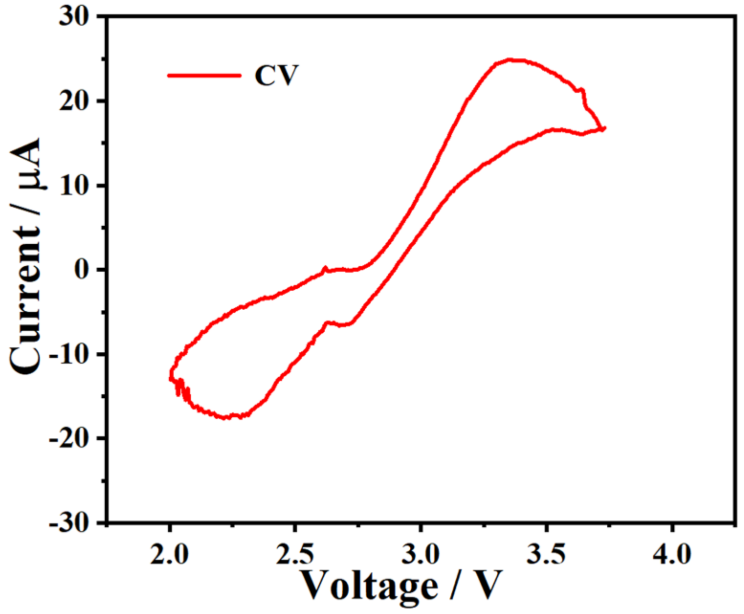

Figure S14. CV curves of Li/LFP cell using liquid metal as current collectors

Table S1. Average strain (failure strain), stress (failure strength) and tensile modulus of PEU-x (x=0, 2, 4, 6)

Sample	PEU-0	PEU-2	PEU-4	PEU-6
Average Strain / MPa	0.062	0.099	0.11	0.13
Average Stress / %	4996	5464	4491	3746
Average Tensile modulus	0.05	0.06	0.04	0.06

Table S2. A comparison between our work and the reported stretchable electrolytes in terms of ionic conductivity, elasticity modulus, and strain at given temperatures.

Ionic conductivity $/10^{-6}$ S cm^{-1}	elasticity modulus /MPa	Strain $/10^{-1}$ mm mm^{-1}	Temperature / K	Reference
120	0	0	298.15	[2]
110	0.8	20	298.15	[2]
0.127	0	0	298.15	[3]
120	0	0	298.15	[4]
226	0	0	298.15	[5]
126	0	0	298.15	[6]
350	0	0	303.15	This work
22	0.11	400	283.15	This work

References

[1] B. Zhou, D. He, J. Hu, Y. Ye, H. Peng, X. Zhou, X. Xie, Z. Xue, *J. Mater. Chem. A* **2018**, *6*, 11725-11733.

[2] D. G. Mackanic, X. Yan, Q. Zhang, N. Matsuhisa, Z. Yu, Y. Jiang, T. Manika, J. Lopez, H. Yan, K. Liu, *Nature Commun.* **2019**, *10*, 1-11.

[3] L. Shi, T. Zhu, G. Gao, X. Zhang, W. Wei, W. Liu, S. Ding, *Nature Commun.* **2018**, *9*.

[4] L. Yu, S. Guo, Y. Lu, Y. Li, X. Lan, D. Wu, R. Li, S. Wu, X. Hu, *Adv. Energy Mater.* **2019**, *9*.

[5] H. Wang, Q. Wang, X. Cao, Y. He, K. Wu, J. Yang, H. Zhou, W. Liu, X. Sun, *Adv. Mater.* **2020**, e2001259-e2001259.

[6] X. Tian, P. Yang, Y. Yi, P. Liu, T. Wang, C. Shu, L. Qu, W. Tang, Y. Zhang, M. Li, *J. Power Sources* **2020**, *450*, 227629.
